# Mineralization at Titanium Surfaces is a Two-Step Process

**DOI:** 10.3390/jfb7010007

**Published:** 2016-03-15

**Authors:** Håkan Nygren, Lars Ilver, Per Malmberg

**Affiliations:** 1 Department of Medical Chemistry and Cell Biology, University of Gothenburg, P.O.B. 420, Göteborg 43050, Sweden; 2 Department of Physics, Chalmers University of Technology, Göteborg 41296, Sweden; lars.ilver@chalmers.se; 3 Department of Chemistry and Chemical Engineering, Chalmers University of Technology, Göteborg 41296, Sweden; malmper@chalmers.se

**Keywords:** titanium, implant, hydroxyapatite

## Abstract

Mapping the initial reaction of implants with blood or cell culture medium is important for the understanding of the healing process in bone. In the present study, the formation of low crystalline carbonated hydroxyapatite (CHA) onto commercially pure titanium (Ti) implants from cell culture medium and blood, is described as an early event in bone healing at implants. The Ti-implants were incubated with cell culture medium (DMEM) or whole blood and the surface concentration of Ca, P and HA was analyzed by XPS, EDX and Tof-SIMS. After incubation with DMEM for 16 h and 72 h, EDX and XPS analysis showed stable levels of Ca and P on the Ti-surface. ESEM images showed an even distribution of Ca and P. Further analysis of the XPS results indicated that CHA was formed at the implants. Analysis with ToF-SIMS yielded high m.w. fragments of HA, such as Ca_2_PO4 at *m*/*z* 174.9 and Ca_3_PO_5_ at *m*/*z* 230.8, as secondary ions at the Ti-surfaces. Analysis of implants incubated in blood for 16 h, with ToF-SIMS, showed initial formation of CHA yielding CaOH as secondary ion. The results indicate that early mineralization at Ti-surfaces is an important step in the healing of implants into bone.

## 1. Introduction

Studies have been made on the structure and formation of the Ti-bone interface [1,2]. Linder *et al.* studying implants in rabbits after 12 weeks of healing reported that the implants were surrounded by mature bone. With TEM, the Ti surface was shown to be covered with an organic layer showing the characteristics of ground substance. Cells at the interface were also separated from the implant by this layer. HA crystals were observed within the ground substance layer, at some spots in contact with the implant. Mineralized bone was present 100–500 nm from the implant surface. The interpretation, made at the time, was that the cells are reacting to the TiO_2_ at the implant surface and the mineral was subsequently secreted by the differentiated osteoblast cells from the bone-side.

The dynamic formation of the established Ti-bone interface as described above has been described by Davies *et al.* The results showed cells separated from the implant surface by an organic layer which stained positive for mineral. This mineral was assumed to be produced by the bone forming cells. The upper layer was partially covered with globular structures. EDX analysis showed the presence of Ca and P in these foci of bone formation.

The main uncertainty tied to the descriptions above is whether or not the HA in the first organic layer was synthesized by bone forming cells or was precipitated from body fluids. Later, several papers have shown that HA forms spontaneously on some TiO_2_ surfaces [3,4,5]. It is also well established that mesenchymal stem cells are differentiated to osteoblasts upon contact with HA [6,7,8,9]. The possible role of HA in healing of Ti-implants then depends on the kinetics of the subprocesses comprising bone healing. The aim of this study is to compare the time dependence of HA precipitation from culture medium and blood with the time dependence of bone formation *in vivo* at Ti-implants, as cited from literature data.

ToF-SIMS analysis of formation of hydroxyapatite at TiO_2_-surfaces indicates that this event precedes the differentiation of stem cells in the kinetics of the formation of the structure of the Ti-bone interface.

## 2. Results and Discussion

**Figure 1:**
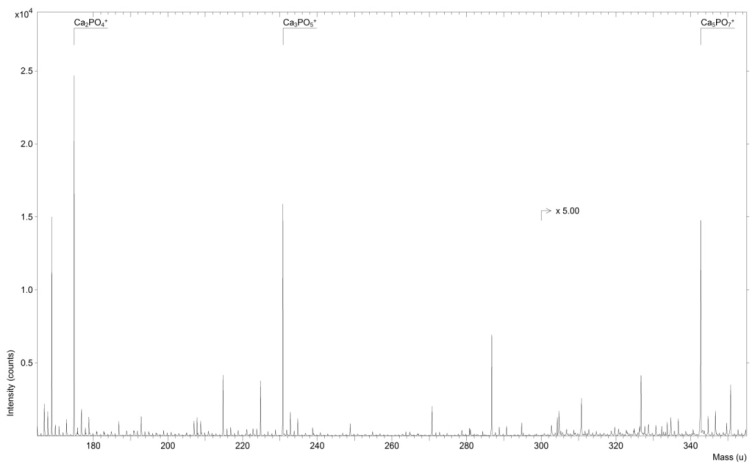
ToF-SIMS spectrum of Ti-implant incubated for 16 h in cell culture medium (DMEM).

Figure 1 shows a ToF-SIMS spectrum of a Ti-implant incubated for 16 h with DMEM.

Peaks representing fragments of HA such as Ca_2_PO_4_ at *m*/*z* 174.9, Ca_3_PO_5_ at *m*/*z* 230.8 and Ca_5_PO_7_ at *m*/*z* 342.7 are marked in the spectrum. These peaks have been defined previously [10,11].

**Figure 2:**
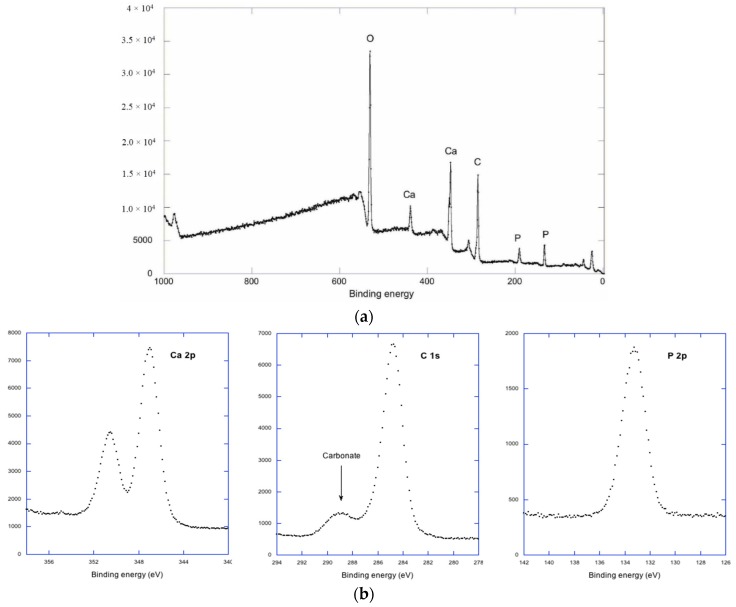
(**a**) Low resolution X-ray photoelectron spectroscopy (XPS) spectrum of Ti-implant incubated 16 h in cell culture medium; (**b**) High resolution XPS spectrum of Ti-implant incubated 16 h in cell culture medium.

Figure 2a shows a low resolution XPS-spectrum for Ti implant sample incubated in DMEM.

The dominating elements are O, Ca, C and P, while the absence of Ti indicates that the substrate is covered by a film, at least 10 nm thick. High resolution spectra are presented in Figure 2b. For Ca and P we have a single component at 347.1 and 133.3 eV respectively. The asymmetry in the P 2*p* is due to an unresolved spin orbit doublet, and the binding energy is typical for phosphate. In the case of C we have a dominating hydrocarbon contamination peak at 284.6 eV and a chemically shifted carbonate peak at 289.2 eV. The analysis program Multi Pak from Physical Electronics (PHI) was used to estimate atomic concentrations of Ca, phosphate P and Carbonate C. The atomic concentrations obtained were Ca 12%, Phosphate P 7.1% and Carbonate C 4.5%. This will give a Ca/P ratio of 1.7, close to the theoretical value for stoichiometric apatite 1.67. Due to an overlay of C from the air, we have slightly underestimated the Ca concentration and the carbonate can be included in the apatite structure without excessive Ca deficiency [12]. A previous study has shown that CHA can be grown from a calcium-containing phosphate buffer on Titania oxide [5]. Recently, it has been shown that HA and CHA differ in their ability to activate stem cells to differentiate into bone-forming cells. Low crystalline CHA exhibited higher osteo-inductivity than HA [9].

**Figure 3:**
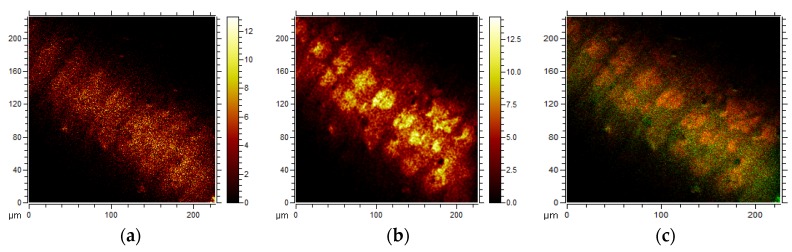
ToF-SIMS images of the (**a**) distribution of cells (phosphocholine (PC), *m*/*z* 184); (**b**) carbonated hydroxyapatite (CaCO_3_ − CO_2_ + H, *m*/*z* 57); (**c**) an overlay of PC (red) and CaOH (green) using high lateral resolution imaging.

Figure 3 shows the results of ToF-SIMS analysis of implants incubated in coagulated blood for 16 h.

No large fragments of HA were found, indicating low surface concentrations of HA at the implants surfaces. The ToF-SIMS analysis has the property of decreasing sensitivity with increasing mass of the secondary ion, which means that low levels of HA can be detected as Ca and PO*_x_* ions [13]. The localization of small fragment (*m*/*z* 57) representing CaCO_3_ − CO_2_ + H, is shown in Figure 3a. This fragment and its polymers have been described previously [10]. The localization of the fragment at *m*/*z* 184 representing phosphocholine, is shown in Figure 3b and an overlay is seen in Figure 3c. The CaCO_3_ is seen evenly distributed over the surface, whereas phosphocholine is localized in circular structures representing cells of surface-adhering coagulated blood [14]. Cell-free areas are covered by plasma proteins and coagulated fibrin as shown previously [14]. These areas also contain CaCO_3_ which is a component of CHA.

**Figure 4:**
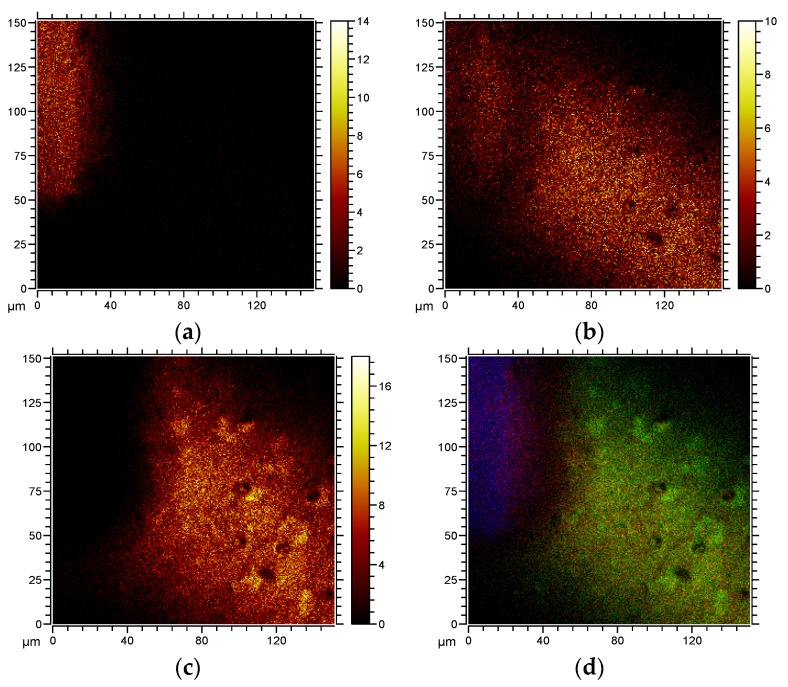
ToF-SIMS images of (**a**) the distribution of Ti (*m/z* 48); (**b**) the distribution of carbonated hydroxyapatite (CaCO3 – CO2 + H, *m/z* 57); (**c**) the distribution of phosphocholine (*m/z* 184), together with (**d**) an overlay image of Ti (blue) CaOH (red) and cells/phoshphocholine (green). The ToF-SIMS data was acquired using high lateral resolution imaging.

Figure 4 shows ToF-SIMS images of the distribution of Ti (*m*/*z* 48.2), CaOH (*m*/*z* 57.37) and phosphocholine (*m*/*z* 184.67), together with an overlay image. A fracture was made in the organic layer, revealing the relation between cells (PC, green), carbonated apatite (CaOH, red) and Ti (blue). The apatite is localized beneath and within the cell layer.

**Figure 5:**
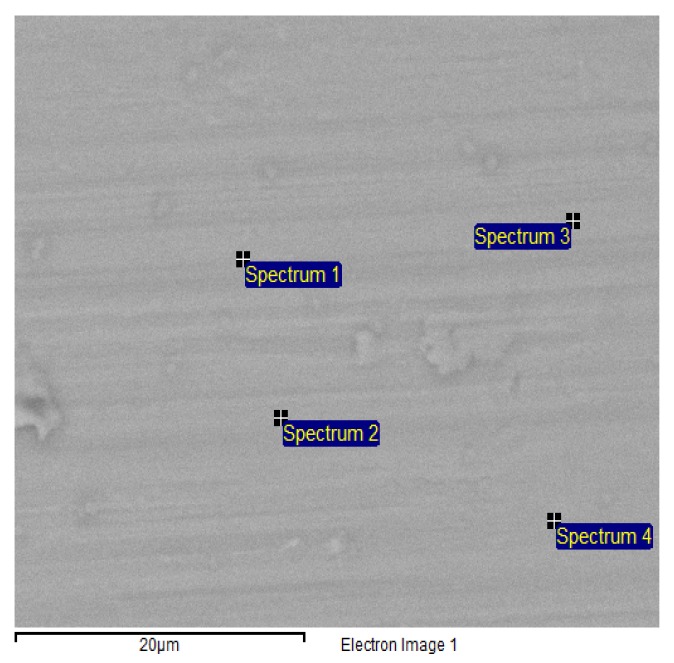
ESEM image of a Ti implant surface after incubation in DMEM for 16 h, using the backscattering detector. EDX spectra were collected at the marked sites.

Figure 5 shows an ESEM image by a backscattering detector of the surface of a titanium implant, incubated in DMEM for 16 h. Backscattering electrons reveal the composition contrast at the surface.

The image shows an even intensity of backscattered electrons, and an absence of large crystals. The apatite layer thus contains low crystalline, CHA. Formation of this apatatite is the first step of the mineralization of the implant.

Studies have been made in order to map significant events in the initial reactions of Ti-surfaces with peripheral blood, lacking mesenchymal stem cells. The first hours of Ti reaction with blood comprises protein adsorption and adhesion and activation of platelets and PMN cells [15,16,17]. Activation of cascade systems, thrombin, kallikrein and complement C5b-9, and their relationship with adherent platelets and polymorph nuclear granulocyte (PMN) activation were also described during the initial Ti-blood reaction. Adhering monocytes were seen after 24–48 h of blood-material contact [18].

The initial healing process of Ti-implants into bone *in vivo* during 1 day through 3 weeks in rat tibiae has also been described [14,19] and cells and tissue on the surface were investigated with regard to bone-forming capacity. Immunofluorescence techniques were used to detect signs of bone formation on hydrophilic and hydrophobic Ti-implant surfaces. Cell viability, alkaline phosphatase (ALP) activity, presence of osteocalcin and cells positive for bone morphogenetic protein-2 (BMP-2) and vascular endothelial growth factor (VEGF) were investigated. The first cells containing BMP-2 were detected after 4 days of healing. VEGF was detected after 8 days on both surfaces. Osteocalcin positive cells were found after 2 weeks. ALP positive cells were found after 8 days, while at 2–3 weeks ALP positive tissue was abundant on both surfaces. Detection of small fragments of CHA was made after 7 days of implantation [13], and bone was seen after 10 days of healing [20]. In conclusion, signs of bone formation, the second step of mineralization of the implant, were detected more than a week after the first step of mineralization occurring after 16 h as shown as shown in the present study.

Thus, the formation of HA, as presented here, is an early event, taking place before osteoblast are seen at the Ti-implant surface (6 days [20]) which means that mesenchymal stem cells arriving at the implant may well be stimulated by HA rather than by the Ti-surface. Since surface-bound hydroxyapatite may act as an activator of mesenchymal stem cells it is interesting to compare the structure of the tissue layers formed on Ti and synthetically made hydroxyapatite surfaces [21]. Deposition of a globular matrix was observed on HA. This was followed by the integration of collagen fibers in this matrix and their subsequent mineralization. An electron-dense layer with a thickness of 20–60 nm was regularly present, containing both organic material and mineralized areas. Thus, a striking similarity between the surface layer formed on Ti-implants and HA-implants can be seen, and it can be justified to conclude that this is due to the formation of HA at the early Ti-blood interface.

The formation of HA at Ti surfaces is more efficient from DMEM than from blood. This finding can be explained by the presence of osteopontin in blood. Osteopontin binds to newly formed HA and inhibits further mineralization [22]. The presence of osteopontin at the bone-Titanium interface has been demonstrated by immune-EM [23]. This HA-protein complex may be an activator of bone forming cells.

## 3. Experimental Section

Titanium samples: Ti- implants, rods with a diameter of 1.5 mm with a smooth surface, were from Elos, Timmersdala, Sweden (Article nr: 557C11872; Lot:X 158128). The purity of all samples was ascertained by EDX, ESCA and ToF-SIMS analysis before making the experiments. Contamination with an organic layer was present on all samples (see Figures S1 and S2, Table S1 in supplementary).

Incubation in fluid: Ti- implants was incubated for 16 and 72 h in 10 mL of DMEM in a humified chamber at 37 °C in cell culture medium (DMEM, Fischer Scientific, Göteborg, Sweden). The volume change of samples was less than 0.1% after 24 h of incubation. The samples were rinsed three times in 50 volumes of distilled water and dried at 60 °C in a ventilated incubator. Ti-implants were also incubated in peripheral blood by immersion of implants in 1 mL of freshly drawn blood in a cell culture dish. The blood was allowed to coagulate for 15 min and 10 mL of DMEM was then added into the dish. Incubation was made for 16 h, followed by rinsing in saline and fixation in 2.5% glutaraldehyde. Finally, the samples were rinsed in distilled water and blown dry in a clean air current.

Analysis: ToF-SIMS analysis was performed with a TOF.SIMS 5 instrument (ION-TOF GmbH, Münster, Germany) using a Bi_3_^+^ cluster ion gun as the primary ion source. Multiple (*n* = 5) regions ranging from 60 μm × 60 μm to 105 μm × 105 μm were analyzed with a pulsed primary ion beam (Bi_3_^+^, 0.24 pA at 25 keV, Dose density 1.12 × 10^11^) with a focus of approximately 2 μm and a mass resolution of *M*/Δ*M* = 5 × 10^3^ fwhm at *m*/*z* 500 as well as using high lateral resolution imaging with a spot size of approx. 200 nm at nominal mass resolution. All spectra were acquired and processed with the Surface Lab software (version 6.3, ION-TOF GmbH, Münster, Germany) and the ion intensities used for calculations were normalized to the total ion dose of each measurement. ToF-SIMS analysis is surface sensitive and detects atoms and molecules in the first nanometer at the surface. ToF-SIMS is not considered a quantitative analysis.

X-ray photoelectron spectroscopy (XPS) was also used for similar untreated and DMEM-incubated samples to characterize the changes in surface chemistry. The XPS spectra were measured (PHI 5000 System, Ulvac, Chigisaki, Japan) using monochromatized Al Ka radiation (hv ¼ 1486.6 eV) as the X-ray source and the photoelectron take-off angle was set at 45. The binding energies were corrected according to the C1*s* peak in CH_2_ (284.6 eV) as the standard.

### Environmental Scanning Electron Microscopy and EDX

An FEI Quanta 200 FEG ESEM (FEI Quanta, Hilsboro, OR, USA) operating at an accelerating voltage of 20 kV was used for imaging and chemical analysis. All images were acquired in the backscattered electron (BSE) imaging mode and at a pressure of 1 torr in the low vacuum region in order to avoid charging effects.

Energy dispersive X-ray (EDX) data was recorded using an Oxford EDX detector (Oxford Instruments Nordic, Lidingö, Sweden) and spectra were evaluated with the INCA software (ETAS Group, Stuttgart, Germany).

## 4. Conclusions

In conclusion, hydroxyapatite forms at the surface of Ti-implants within hours, both from culture medium and blood. Blood factors inhibit the spontaneous growth of hydroxyapatite in blood. This process occurs more rapidly than the recruitment of stem cells to the area, and an osteopontin-HA complex is probably the initiator of bone formation.

## Figures and Tables

**Table:** 

Spectrum	C	O	P	Ca	Ti	Total
Spectrum 1	4.73	14.30	0.40	0.49	80.08	100.00
Spectrum 2	4.85	14.60	0.37	0.54	79.64	100.00
Spectrum 3	4.96	14.38	0.38	0.55	79.73	100.00
Spectrum 4	4.68	14.81	0.39	0.56	79.57	100.00
Mean	4.80	14.52	0.39	0.53	79.75	100.00
Std. deviation	0.13	0.23	0.01	0.03	0.22	–

## Supplementary Materials

Supplementary materials can be accessed at: http://www.mdpi.com/2079-4983/7/1/7/s1.
