# Supplementary material for: Mineralization at Titanium Surfaces is a Two-Step Process
Source: J Funct Biomater. 2016 Mar 15;7(1):7. doi: 10.3390/jfb7010007 (PMC4810066; doi:10.3390/jfb7010007)
Supplement: Supplementary file 1 [file jfb-07-00007-s001.pdf]

Journal of
Functional
Biomaterials

Supplementary Materials

Characterisation of implants manufacturing (Figure S1).

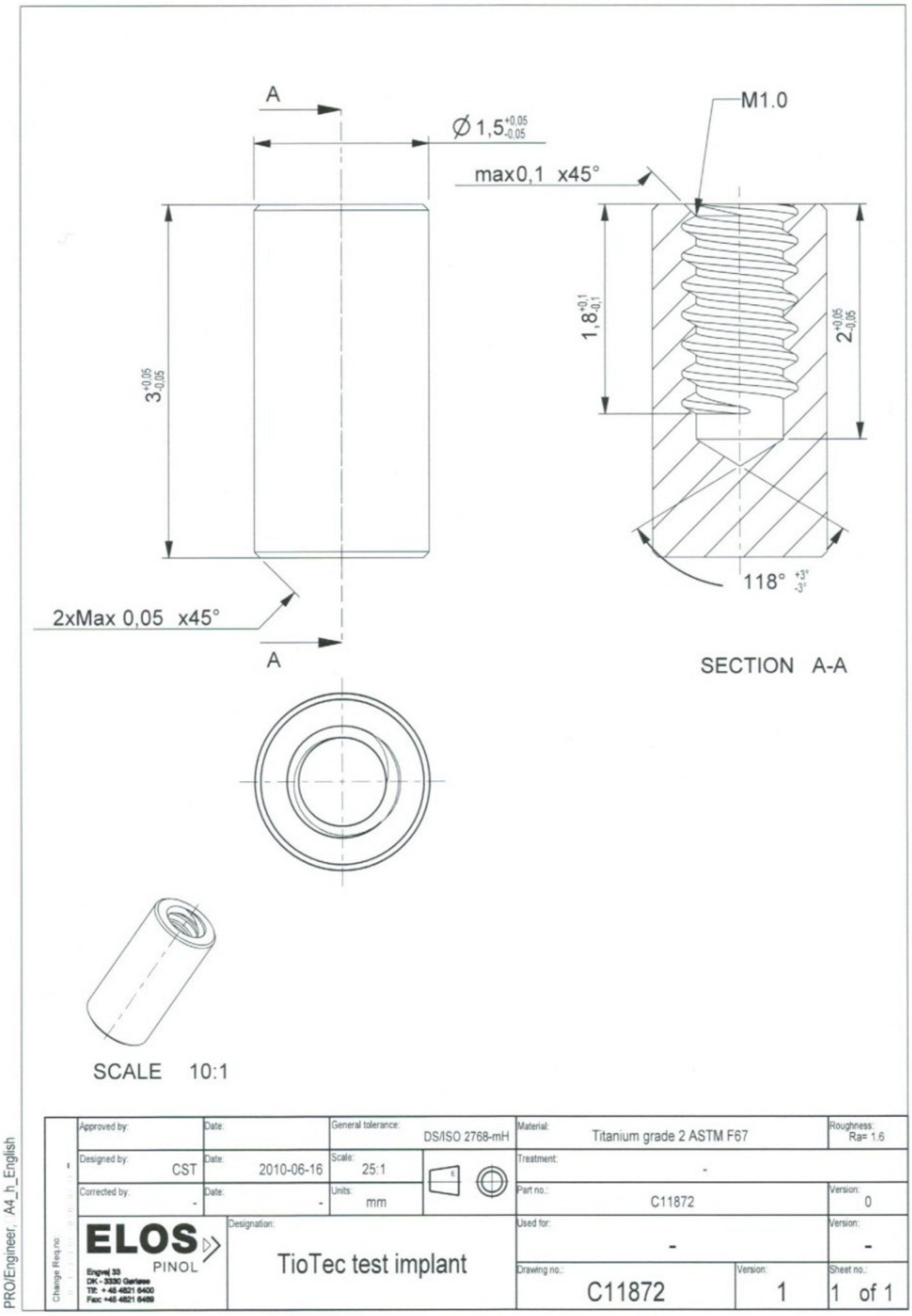

Figure S1. Titanium implant (commercially available).

Scanning electron microscopy of a Ti-implant taken directly from the manufacturers package. Marks of the milling tool can be seen in the micrometer range (Figure S2).

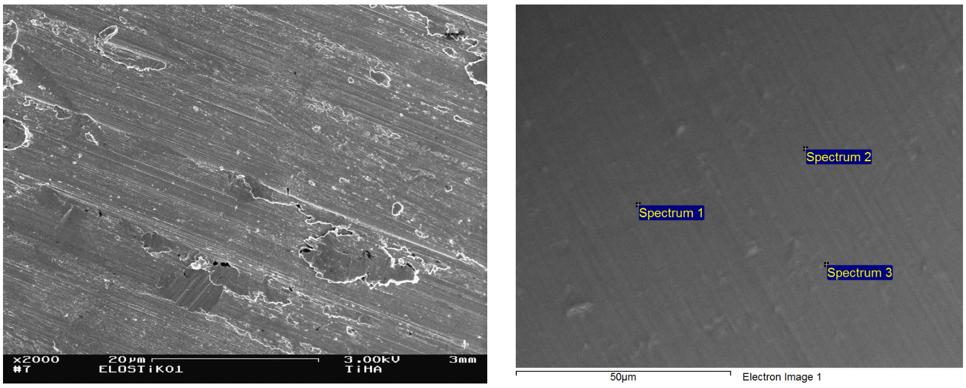

(**a**) (**b**)

Figure S2. (**a**) ESEM image and (**b**) EDX analysis of the c.p. Ti-implant.

The EDX data (Table S1) indicate contamination with a carbon layer.

Table S1. Atomic species analysed with EDX.

Ti, c.p.	C	O	Ti
Spectrum 1	28.87	8.76	62.22
Spectrum 2	29.10	9.28	61.42
Spectrum 3	26.56	–	73.26

© 2016 by the authors; licensee MDPI, Basel, Switzerland. This article is an open access article distributed under the terms and conditions of the Creative Commons by Attribution (CC-BY) license (http://creativecommons.org/licenses/by/4.0/).
